# Genome characterization of *Streptomyces* sp. strain VNUA74, a potential biocontrol against pathogenic fungus *Colletotrichum* spp.

**DOI:** 10.1128/MRA.00873-23

**Published:** 2023-11-14

**Authors:** Thu Thi Nguyen, Son Truong Dinh, Canh Xuan Nguyen

**Affiliations:** 1 Department of Microbial Biotechnology, Faculty of Biotechnology, Vietnam National University of Agriculture, Hanoi, Vietnam; 2 Department of Plant Biotechnology, Faculty of Biotechnology, Vietnam National University of Agriculture, Hanoi, Vietnam; 3 Department of Applied Biotechnology, Institute of Agrobiology, Vietnam National University of Agriculture, Hanoi, Vietnam; Rochester Institute of Technology, Rochester, New York, USA

**Keywords:** *Streptomyces*, *Colletotrichum*

## Abstract

The whole genome sequence of *Streptomyces* sp. strain VNUA74, isolated from soil in a banana farm in Vietnam and exhibited fungicidal effects on banana *Colletotrichum* spp., was sequenced by PacBio RS II and DNBseq sequencing platforms. The complete circular genome is 7,250,076 bp with a GC content of 72.69%.

## ANNOUNCEMENT

**Fig 1:**
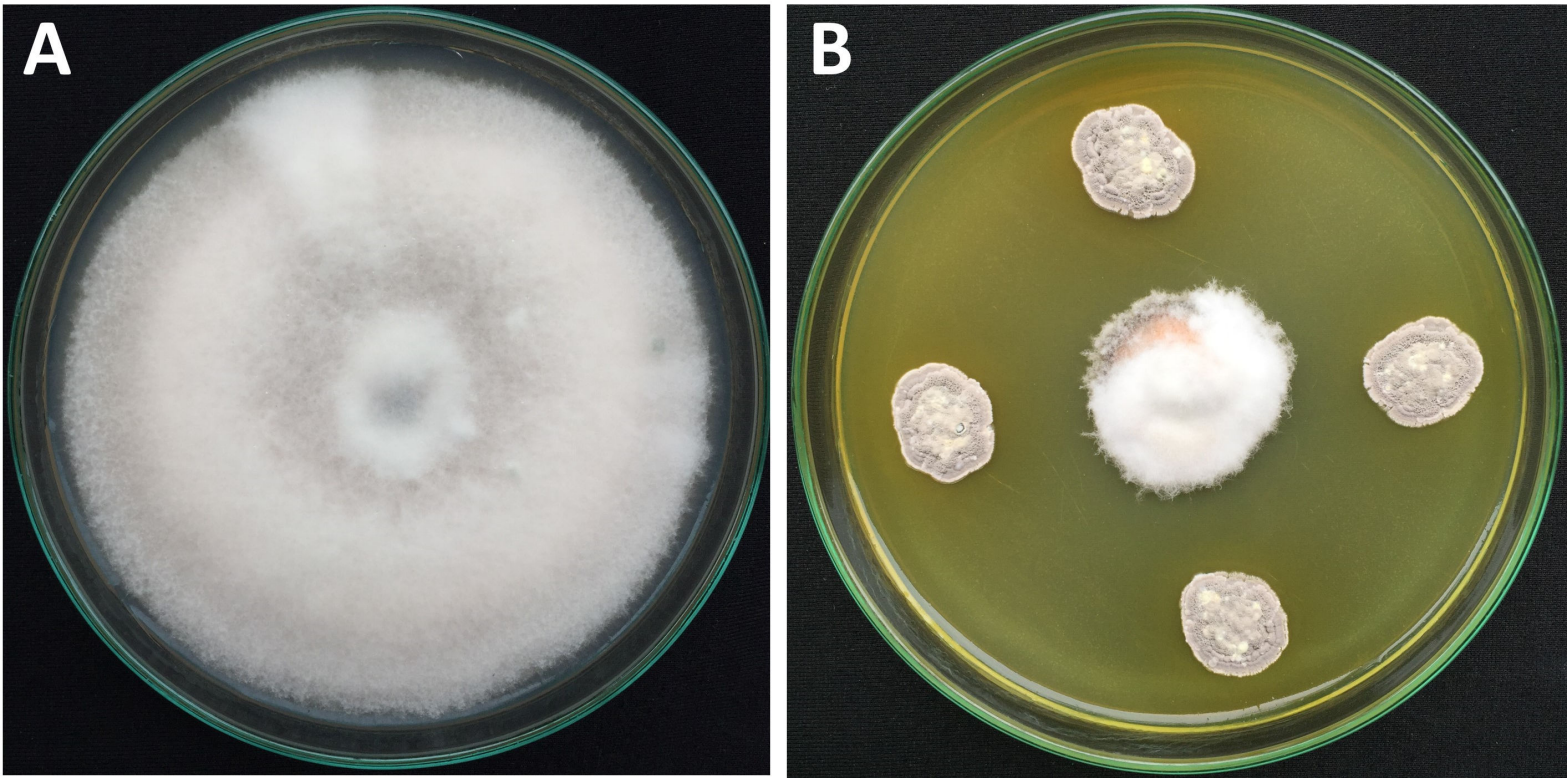
Fungistatic activities of *Streptomyces* sp. strain VNUA74 on the mycelial growth of *Colletotrichum* sp. (**A**) Growth of *Colletotrichum* sp. on control medium. (**B**) The inhibitory effect of mycelium growth of *Colletotrichum* sp. by *Streptomyces* sp. strain VNUA74.

The *Streptomyces* sp. VNUA74 strain was isolated from banana rhizosphere soil collected at Hung Yen province, Vietnam (1). The strain exhibited fungistatic activity against *Colletotrichum* spp. (Fig. 1) (2).

VNUA74 strain grown on Gause’s No. 1 liquid medium for 2 days was used for DNA isolation using a modified procedure [Phenol:Chloroform:Isoamyl alcohol (25:24:1) extraction was added before Chloroform:Isoamyl alcohol] (3). The integrity and purity of extracted DNA were confirmed by gel electrophoresis and spectrophotometer (3) before sequencing at the Beijing Genomics Institute, Shenzhen, China.

On the DNBSEQ platform, 1 µg genomic DNA was randomly fragmented. The fragments between 200 and 400 bp, selected by Agencourt AMPure XP-Medium kit, were used to synthesize the single-strand circle DNA library. The qualified libraries were sequenced by BGISEQ-500 sequencer for paired-end 2 × 150 bp sequencing following the company’s protocol (4). SOAPnuke v1.5.5 was used to remove low quality reads and adaptors before assembly (https://github.com/BGI-flexlab/SOAPnuke) (5). The DNBSEQ sequencing obtained 7,685,154 paired-end sequence reads and 158.0× coverage.

On the PacBio platform, the DNA was prepared with Covaris g-TUBE to the correct size (about 10–15 kb), then the two ends of selected DNA fragments were joined to form a dumbbell structure using the SMRT bell template prep kit v1.0 (Pacific Bioscience). The PacBio subreads less than 1 kb long were removed. The PacBio sequencing obtained 278,519 subreads, 2,269,824,271 bp, and 313.0× coverage. The N50 and N90, subreads max length, and subreads mean length values are 8,738 bp, 5,919 bp, 191,936 bp, and 8,149 bp, respectively. Both Canu v1.5 (estn = 24, npruseGrid = 0, corOvlMemory = 4; https://github.com/marbl/canu/releases) and Falcon software were used to correct the subreads and assembly and then the best assembly result was chosen (6). The genome was then polished by GATK v1.6–13 (-cluster 2 -window 5 -stand_call_conf 50 -stand_emit_conf 10.0 -dcov 200 MQ0 ≥4; https://www.broadinstitute.org/gatk/) (7). The assembled genome was circularized by Circlator v1.5.5 (8). The prediction of protein-coding genes was done using Glimmer 3.02 software (http://www.cbcb.umd.edu/software/glimmer/) with Hidden Markov models (-o * -g * -t * -l linear) (9, 10). For tRNA, rRNA, and small RNA (sRNA) identification, the RNAmmer v1.2, tRNAscan-SE v1.3.1 (–s Species –m Type –gff *. rRNA.gff –f *.rRNA.fq) (11), and the Rfam v9.1 database were employed (–*P* blastn –W 7 –e 1 –v 10000 –b 10000 –m 8 –i subfile –o *.blast.m8) (12).

The *Streptomyces* sp. strain VNUA74 genome has a single circular chromosome with 7,250,076 bp, G + C content of 72.69%, and 6,727 predicted genes. Genome annotation resulted in a total of 65 tRNAs, 18 rRNAs, and 15 sRNA with an average length of 83.6 bp. There are 973 minisatellite DNA and 93 microsatellite DNA with repeat size rank from 15 to 63 bp and 3 to 10 bp, respectively. In addition, there are seven incomplete prophages that were predicted using the PhiSpy, version:3.7.8 (https://github.com/linsalrob/PhiSpy; default settings) (13). CRISPRCasFinder version:4.2.19 (https://crisprcas.i2bc.paris-saclay.fr/CrisprCasFinder/; default settings) (14) was used to uncover 10 CRISPR sequences.

## Data Availability

The whole-genome sequence and associated data have been deposited at GenBank (accession number CP133215.1) and the Sequence Read Archive (SRR25325810 and SRR25325811).
